# Supplementary material for: The Quality of Life and Associated Factors Among Older Adults in Central Nepal: A Cross-Sectional Study Using the WHOQOL-OLD Tool
Source: Int J Environ Res Public Health. 2025 Apr 27;22(5):693. doi: 10.3390/ijerph22050693 (PMC12111222; doi:10.3390/ijerph22050693)
Supplement: Supplementary file 1 [file ijerph-22-00693-s001.zip › ijerph-3545605-supplementary.pdf]

Supplementary Materials:

Table S1. Correlation among six domains of WHOQOL-OLD.

	Sensory abilities	Autonomy	Past, present and future activities	Social participation	Death and dying	Intimacy
**Sensory abilities**	1					
**Autonomy**						
Correlation coefficient	−0.328**	1				
p-value	<0.001					
**Past, present and future activities**						
Correlation coefficient	−0.104*	0.578**	1			
p-value	0.046	<0.001				
**Social participation**						
Correlation coefficient	−0.210**	0.531**	0.645**	1		
p-value	<0.001	<0.001	<0.001			
**Death and dying**						
Correlation coefficient	0.260**	−0.319**	−0.236**	−0.224**	1	
p-value	<0.001	<0.001	<0.001	<0.001		
**Intimacy**						
Correlation coefficient	−0.168**	0.448**	0.421**	0.424**	−0.231**	1
p-value	0.001	<0.001	<0.001	<0.001	<0.001	

** Correlation is significant at the 0.01 level. *Correlation is significant at the 0.05 level.

Table S2. Overall quality of life score by different variables using ANOVA test.

Variables	Mean	SE	*p*-value
**Age**			0.124
60–69	75.09	0.64	
70 and above	73.82	0.53	
**Gender**			**<0.001**
Male	75.80	0.54	
Female	72.62	0.59	
**Ethnicity**			0.213
Upper caste	74.69	0.45	
Janajati	73.27	1.12	
Dalit	71.87	2.0	
**Religion**			**0.581**
Hindu	74.30	0.43	
Others*	75.05	1.19	
**Marital status**			**<0.001**
Married	75.24	0.50	
Unmarried*	72.22	0.65	
**Type of family**			0.601
Nuclear	73.97	1.03	
Joint	74.49	0.44	
**Living with**			0.214
Son	74.65	0.44	
Spouse	74.06	1.41	
Daughter *	71.92	1.47	
**Education**			**<0.001**

Illiterate	72.79	0.45	
Literate	77.67	0.75	
**Household income**			**0.001**
<40,000	72.26	0.63	
≥40,000	76.08	0.51	
**Employment status**			0.893
Employed	74.57	1.39	
Unemployed/ retired	74.36	0.43	
**Personal income**			0.552
No	73.80	1.03	
Yes	74.48	0.44	
**Food security**			**0.044**
Less than 6 months	71.05	2.05	
Six or more months	74.58	0.41	
**Alcohol consumption**			**0.015**
No	74.02	0.43	
Yes	77.09	1.22	
**Tobacco use**			0.232
No	74.09	0.48	
Yes	75.23	0.75	
**Chronic disease**			0.672
No	74.16	0.70	
Yes	74.51	0.50	
**Family support**			0.089
No	70.00	2.04	
Yes	74.49	0.41	
**Daily work support**			0.627
No	74.88	1.25	
Yes	74.30	0.43	
**Emotional support**			**0.796**
No	74.54	0.76	
Yes	74.31	0.48	
**Decisional support**			0.777
No	74.19	0.77	
Yes	74.45	0.48	
**Economic support**			0.954
No	74.43	0.93	
Yes	74.37	0.45	
**Accessibility**			**0.001**
No accessibility	68.84	2.17	
Accessibility	74.68	0.41	
**Availability**			**<0.001**
No availability	76.14	0.49	
Availability	72.17	0.64	
**Affordability**			**<0.001**
No affordability	70.93	1.01	
Affordability	75.34	0.42	
**Physical activity**			**<0.001**
No	72.48	0.58	
Yes	75.85	0.54	

Others*: Buddhist,/Muslim; Unmarried* denotes Unmarried/separated/widow/widower; Business* denotes Business/ House/Rent/Agriculture/Writer/Driver; ANOVA: Analysis of Variance. SE: Standard Error
